# Time-of-Flight MRA of Intracranial Aneurysms with Interval Surveillance, Clinical Segmentation and Annotations

**DOI:** 10.1038/s41597-024-03397-8

**Published:** 2024-05-30

**Authors:** Chloe M. de Nys, Ee Shern Liang, Marita Prior, Maria A. Woodruff, James I. Novak, Ashley R. Murphy, Zhiyong Li, Craig D. Winter, Mark C. Allenby

**Affiliations:** 1 School of Chemical Engineering, The University of Queensland, Brisbane, Australia; 2 Herston Biofabrication Institute, The Royal Brisbane and Women’s Hospital, Brisbane, Australia; 3 Centre for Clinical Research, Faculty of Medicine, The University of Queensland, Brisbane, Australia; 4 Department of Medical Imaging, The Royal Brisbane and Women’s Hospital, Brisbane, Australia; 5 School of Mechanical, Medical and Process Engineering, Queensland University of Technology, Brisbane, Australia; 6 School of Architecture, Design and Planning, The University of Queensland, Brisbane, Australia; 7 Kenneth G Jaimieson Department of Neurosurgery, The Royal Brisbane and Women’s Hospital, Brisbane, Australia

**Keywords:** Brain, Brain imaging, Magnetic resonance imaging, Cerebrovascular disorders, Aneurysm

## Abstract

Intracranial aneurysms (IAs) are present in 2–6% of the global population and can be catastrophic upon rupture with a mortality rate of 30–50%. IAs are commonly detected through time-of-flight magnetic resonance angiography (TOF-MRA), however, this data is rarely available for research and training purposes. The provision of imaging resources such as TOF-MRA images is imperative to develop new strategies for IA detection, rupture prediction, and surgical training. To support efforts in addressing data availability bottlenecks, we provide an open-access TOF-MRA dataset comprising 63 patients, of which 24 underwent interval surveillance imaging by TOF-MRA. Patient scans were evaluated by a neuroradiologist, providing aneurysm and vessel segmentations, clinical annotations, 3D models, in addition to 3D Slicer software environments containing all this data for each patient. This dataset is the first to provide interval surveillance imaging for supporting the understanding of IA growth and stability. This dataset will support computational and experimental research into IA dynamics and assist surgical and radiology training in IA treatment.

## Background & Summary

Intracranial aneurysms (IAs) are an outpouching of the arterial wall, caused by a weakening and degradation of arterial tissue layers^1–3^. IAs are present in 2–6% of the global population, and their rupture causes 85% of subarachnoid haemorrhages with a 30–50% mortality rate^4–7^. IAs are also a leading cause of stroke in adults below 65 years of age^8,9^.

Intra-arterial digital subtraction angiography (DSA), computed tomography angiography (CTA) and time-of-flight magnetic resonance angiography (TOF-MRA) are widely implemented for clinical identification of IAs^10^. TOF-MRA is less invasive and thus more frequently used as it does not require the use of an intravenous contrast material^10^. Although, some radiologists may still administer contrast material to enhance image quality.

With the widespread use of these imaging modalities for a variety of clinical presentations, the incidental identification of IAs is increasing^11,12^. However, IA detection relies on specialised radiologists or neuroradiologists, and as implementation of these imaging modalities increases so-to does the workload of neuroradiologists, making it challenging to reach reporting demand on all cranial angiograms^13,14^. In addition, these specialised radiologists are rarely available outside of specialised facilities and in rural areas, reducing rates of detection.

Furthermore, once an IA has been detected, clinicians are currently reliant on experience and known patient risk factors to determine whether surgical treatment is required. Existing methods to evaluate rupture risk include use of the PHASES risk prediction score (based on Population, Hypertension, Age, Size, Earlier haemorrhage and Site of IA) and the unruptured intracranial aneurysm treatment score (UIATS) which in some cases remain insufficient in providing a clear recommendation for treatment^15–17^.

To improve IA detection and our understanding of pathophysiology and rupture tendency, various *in silico* and *in vitro* models have been developed. These include, but are not limited to: the development of automated algorithms to aid clinicians in detecting aneurysms^5,12,13^; evaluation of the effect of haemodynamic stress through computational fluid dynamics (CFD) models^18,19^; and investigating cell behaviour in *in vitro* aneurysm models with patient-specific geometries^20–22^.

However, many of these studies are limited to analysis of small patient cohorts (n < 15) and/or do not provide their datasets for public use^14^. While several publicly available datasets of healthy brain vasculature exist, including the IXI (http://brain-development.org) database, there is a lack of large and publicly available datasets with IAs, with none containing patients with interval surveillance imaging. This hinders research efforts into IA detection and eventual rupture by limiting collaboration, evaluation, validation, and reproducibility of *in silico* and *in vitro* models. Additionally, given the multitude of IA morphologies that can arise and inter-individual variability, the generation of large publicly available datasets with expert clinical measurements and segmentations are imperative for a complete understanding of risk factors related to IA formation and rupture.

The AneurRisk Project (http://ecm2.mathcs.emory.edu/aneuriskweb/index) thus arose in 2008 to unite researchers in the goal to uncover the complex pathology around aneurysms, providing DSA scans of 65 patients with IAs. More recently, Di Noto *et al*.^14^ published an open-access dataset comprising 284 subjects, with 127 healthy controls and 157 patients with 198 aneurysms. They demonstrated the use of this dataset in developing an automated deep learning algorithm to detect IAs^14^. To the best of our knowledge, this is currently the only TOF-MRA aneurysm dataset available.

To further support this effort to address bottlenecks in data availability, we provide an open-access annotated TOF-MRA dataset comprising 63 subjects containing IAs. Importantly, this work differs to the existing databases by providing interval surveillance imaging on 24 subjects, ranging from 1–12 years of surveillance. This data is useful for investigating changes in size and morphology of IAs over time, which may help validate the predictive risk of IA geometry and fluid dynamics in rupture. These surveillance images could also highlight changes in vessel geometry and fluid dynamics after treatment to inform surgical planning and aid in the development of new treatment options.

**Fig. 1:**
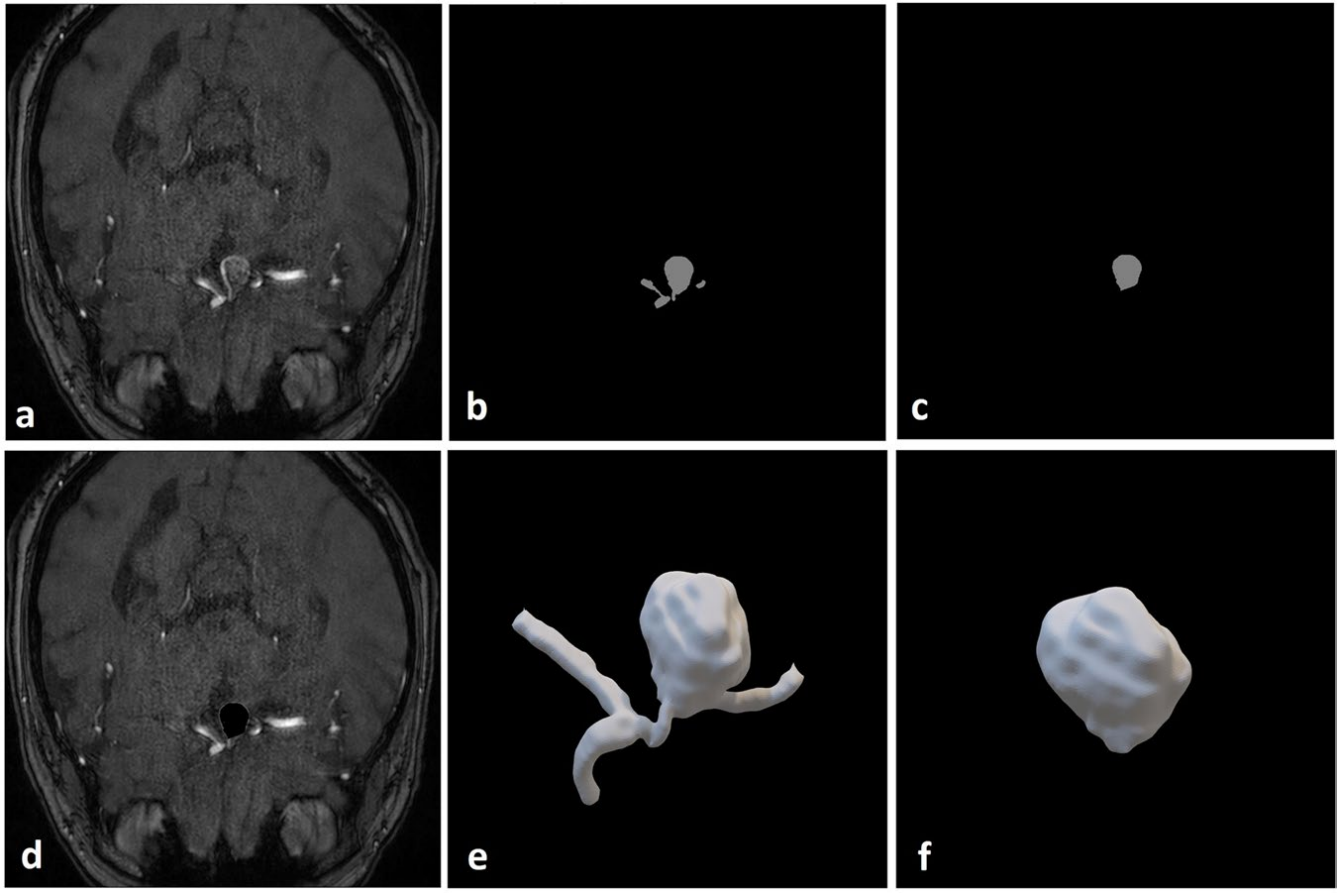
One transverse cross-section of a Time-Of-Flight Magnetic Resonance Angiography (TOF-MRA) scan for one representative patient (sub-042) with a right A1/anterior communicating artery (ACOM) aneurysm with clinician-annotated size of 12 × 10 × 12 mm. (**a**) High-resolution TOF-MRA; (**b**) Threshold-segmented mask of aneurysm with parent artery; (**c**) Threshold-segmented mask of aneurysm only; (**d**) TOF-MRA with aneurysm overlay in black; (**e**) 3D model of aneurysm with parent vessel smoothed using Taubin’s algorithm in 3D Slicer; (**f**) 3D model of aneurysm smoothed using Taubin’s algorithm in 3D Slicer. Brightness for (**a**) and (**d**) were linearly adjusted.

We also provide IA characteristics including size and location, with 3D aneurysm models in standard tessellation language (STL) file format, clinical annotations, labelled maps with the IA and parent vessels, and 3D slicer files comprising all masks within a single workspace (one example patient dataset depicted in Fig. 1). This dataset will therefore support ongoing collaborative research to ultimately improve clinical detection, understanding and treatment of IAs.

## Methods

### Data overview

**Fig. 2:**
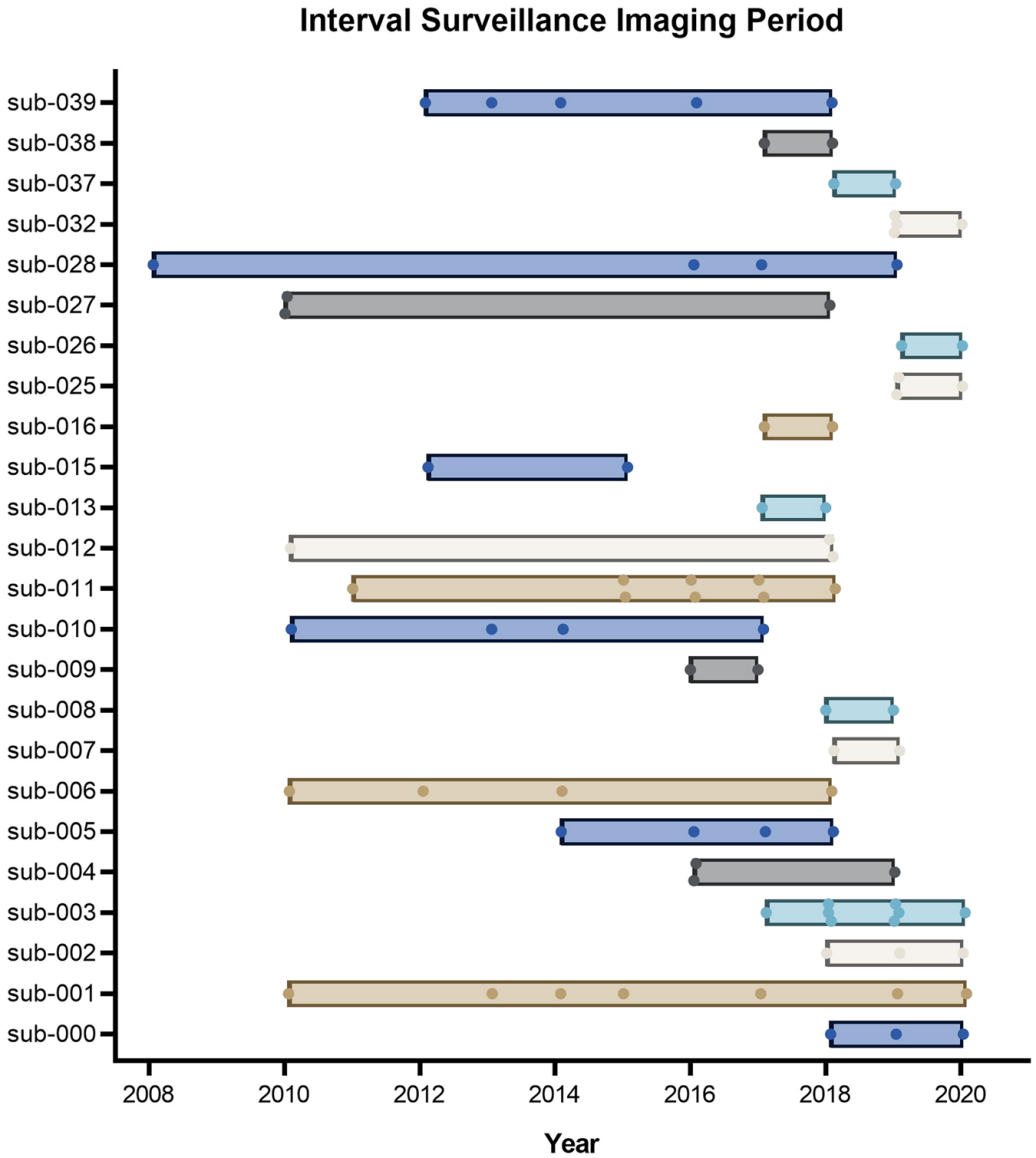
Imaging period for patients with interval surveillance (n = 24). Each data point represents one imaging session. Sub-008, sub-009 and sub-013 each had surveillance images taken within one year of the first appointment.

The TOF-MRA scans were acquired through Metro North Hospital and Health Service (MNHHS) at various institutions between 2008 and 2022, totalling 63 de-identified patients. Interval surveillance imaging was conducted on 24 of the 63 patients through follow-up appointments, as depicted in Fig. 2. The research protocol used for de-identified data collection and provision in a public open-access repository under a waiver of patient consent was approved by the Royal Brisbane and Women’s Hospital (RBWH) Human Research Ethics Committee (Ref: LNR/2019/QRBW/49363) and the Queensland Department of Public Health (Ref: Public Health Agreement #49363).

### Image acquisition

MR images were acquired using GE, Philips and Siemens MR scanners with varying technical specifications and acquisition parameters, as summarised in Table 1. In some cases, longitudinal imaging was conducted on different MR scanners across the surveillance period. These details are provided in the supporting Excel spreadsheet, ‘*Clinical Annotations_MR Specifications_Demographics_Image Quality Analysis*’ uploaded with the dataset for each individual scan.

**Table 1:** Magnetic Resonance scanners, technical specifications and acquisition parameters of TOF-MRA scans. RT = Repetition time, TE = time to echo.

Vendor	Model	# of Scans	Avg. Field Strength [T]	Avg. RT [ms]	Avg. TE [ms]	Avg. Voxel Spacing [mm^3^]
GE	Discovery MR750w	1	3	24.0	5.7	1.00 × 1.00 × 0.50
	Signa HDxt	3	1.5	22.0	3.5	1.20 × 1.16 × 0.60
	Unknown	3	2	28.0	3.8	1.40 × 1.36 × 0.70
Philips	Ingenia	25	3	18.6	3.5	0.66 × 0.91 × 0.82
	Unknown	6	3	21.7	3.5	1.13 × 1.10 × 0.57
Siemens	Avanto	8	1.5	164.7	6.6	2.04 × 1.94 × 15.56
	Magnetom Vida	19	3	24.1	4.0	0.41 × 0.41 × 0.42
	Skyra	12	3	21.2	3.6	0.44 × 0.44 × 14.66
	TrioTim	26	3	22.7	4.1	0.48 × 0.48 × 20.49
	Unknown	34	3	22.7	4.0	0.47 × 0.47 × 7.53
	Verio	6	3	22.0	3.7	0.49 × 0.49 × 18.63

Data was then de-identified by radiologists using PixelMed^TM^ DicomCleaner^TM^ to remove all health information identifiers. De-identified data was provided for research purposes through established governance processes at the RBWH and data distributed to researchers as per data sharing agreements approved by the Queensland Department of Public Health (Ref: Public Health Agreement #49363). Scans were further cropped to remove the front portion of the skull and facial features while maintaining visibility of the Circle of Willis, with all data visually inspected to ensure appropriate de-identification before release.

Scans were acquired in DICOM (Digital Imaging and Communications in Medicine) format and converted to NIFTI (Neuroimaging Informatics Technology Initiative) files. Where files contained multiple scanning sequences, these were stripped to comprise only de-identified TOF-MRA scanning sequences for each patient.

### Segmentation and annotation

Angiographic image sequences were imported into 3D Slicer, with segmentation and annotation of aneurysms and parent vessels performed by a neuroradiologist. Aneurysms were first manually detected by scrolling through the slices of the scan, and threshold segmentation applied to extract the vascular tree. The aneurysm and parent vessels were then isolated by cropping the larger vascular tree. Some aneurysms that were too large or contained clots required manual segmentation of focal areas due to the lack of contrast. Voxel-wise annotations of the aneurysm and parent vessels were then created, where the neuroradiologist manually delineated the aneurysm from the parent vessel, guided by changes in vessel curvature. Annotations were smoothed using Taubin’s algorithm in 3D Slicer, and surface meshes produced using the Flying Edges algorithm in 3D Slicer before exporting as 3D STL mesh files. Original segmentations and binary label maps are provided as raw voxel-wise annotations, allowing the user of the dataset to apply different smoothing settings as necessary.

## Data Records

63 patients with 85 IAs were imaged using TOF-MRA. 39 patients comprise singular scans while 24 patients include interval surveillance images. Patient images were organised according to the Brain Imaging Data Structure (BIDS) and were made publicly available through the OpenNeuro repository 10.18112/openneuro.ds005096.v1.0.0^23^.

All information relating to MR acquisition parameters, clinical annotations and patient age and gender has been uploaded in the supporting Excel spreadsheet, ‘*Clinical Annotations_MR Specifications_Demographics_Image Quality Analysis*’ uploaded with the dataset. A summary of aneurysm characteristics is provided in Table 2, with location and size grouped according to the PHASES score due to its clinical use.

**Table 2:** Summary of patient scans, aneurysm locations and size grouped according to the PHASES score currently used for clinical interpretation. ICA = Internal Carotid Artery, MCA = Middle Cerebral Artery, ACA = Anterior Cerebral Arteries, Pcom = Posterior Communicating Artery, Posterior = posterior circulation (including vertebral, basilar, cerebellar, and posterior cerebral arteries).

		Count
**Total Patients**		63
**Patient Scans**	Patients with 1 aneurysm	47
	Patients with > 1 aneurysm	16
	Patients with single scans	39
	Patients with follow-up scans	24
**Aneurysm Location**	MCA and M1	30
	ICA	24
	ACA/Pcom/Posterior	31
**Aneurysm Maximum Diameter**	<7.0 mm	46
	7–9.9 mm	23
	10–19.9 mm	10
	>20 mm	2
	Difficult or previously treated aneurysm	4

**Fig. 3:**
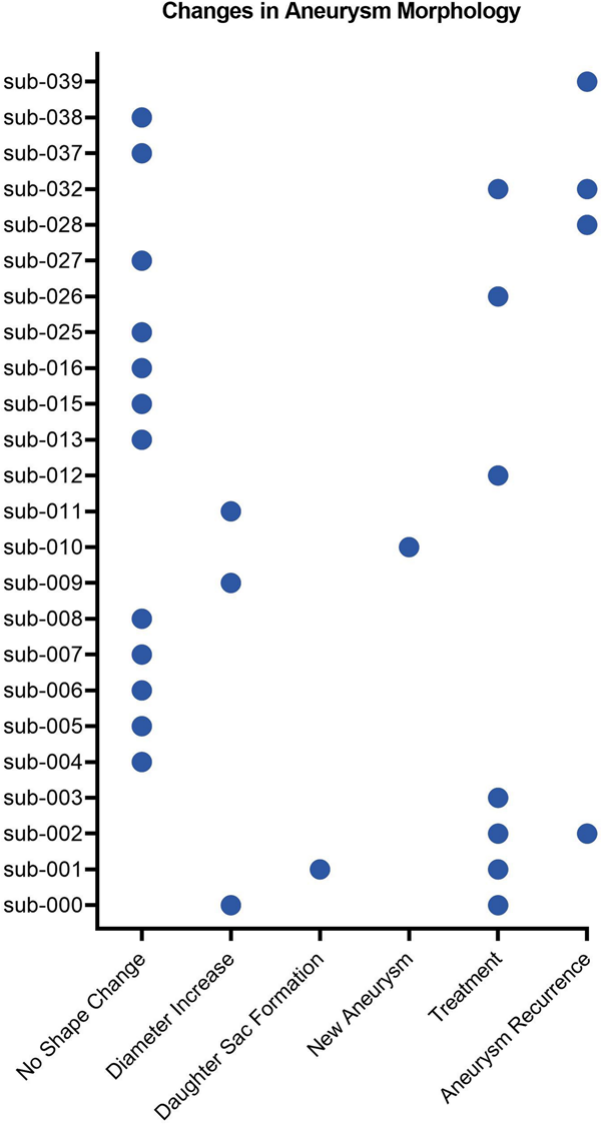
Summary of changes in aneurysm morphology across subjects with interval surveillance imaging. A ‘diameter increase’ was indicated if an aneurysm exhibited more than a 2 mm increase. A ‘new aneurysm’ indicates initiation of a new aneurysm at a different location during imaging. ‘Treatment’ refers to scans taken before- and after- aneurysm treatment. Aneurysms that have recurred after treatment are also indicated.

Patients having undergone interval surveillance imaging have been further analysed for increases in size, daughter sac formation, aneurysm recurrence, new aneurysm formation, and treatment. This has been summarised in Fig. 3 below. Importantly, due to the poor spatial resolution offered by TOF-MRA scans, a shape change is only indicated for an increase in diameter of more than 2 mm. If aneurysm shape change is suspected from a TOF-MRA in the clinic, subsequent CTA or DSA is typically performed to provide more accurate analyses of aneurysm morphology and confirm any suspected shape change.

**Fig. 4:**
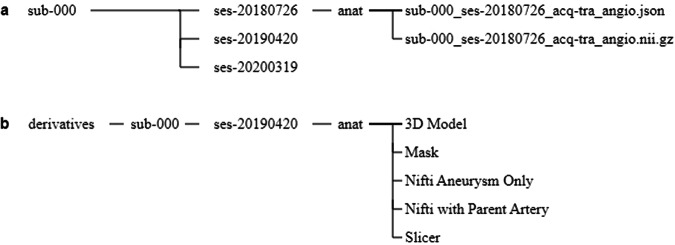
Folder structure of the dataset for sub-000. (**a**) Original Time-of-Flight Magnetic Resonance Angiography (TOF-MRA) scans are provided for each individual subject with all scanning sessions available. (**b**) A derivatives folder provides all segmentations and annotations performed for a single scanning session for each subject. For sub-000, the TOF-MRA from ses-20190420 was used for segmentation and annotation by a neuroradiologist as this was the most recent high-quality scan conducted before aneurysm coiling.

Each patient comprises at least one angiography scan in NIFTI format with accompanying JSON (JavaScript Object Notation) file organised according to the BIDS standard (Fig. 4a). Some patients include multiple angiographic scans for a single scanning session, with some scans including both single or multi-slab imaging, or with varying resolution. These have been indicated through corresponding acquisition labels (e.g. ‘acq-singleslab’ for single slab imaging or ‘acq-highres’ for high resolution).

In addition to the original patient data, segmentations and annotations performed by a neuroradiologist using 3D Slicer are provided for a single scanning session. Where several scanning sessions are available for 24 patients, segmentations were performed on scans before treatment if applicable, on the best quality scan, or on the most recent scan provided as determined by the neuroradiologist. These annotations are contained in a derivatives folder using a similar folder structure to the BIDS specification.

Specifically, for each patient a mask has been created for the segmented aneurysm and parent vessels (Fig. 4b – ‘Nifti Aneurysm Only’ and Fig. 4b – ‘Nifti with Parent Artery’ respectively) overlayed onto the original NIFTI scan (Fig. 4b – ‘Mask’), along with the segmented 3D STL aneurysm models (Fig. 4b – ‘3D Model’), and the 3D Slicer scene where segmentation and annotations were performed (Fig. 4b – ‘Slicer’). For patients where characterisations and annotations were performed using DSA or CTA scans, only the segmented masks and 3D STL models are provided of the aneurysm, parent vessels and vascular tree.

An example of the data provided for a single patient is summarised in Fig. 4.

## Technical Validation

Segmented aneurysm morphology and resulting CFD analyses can be influenced by imaging technique and image quality, as well as segmentation and post-processing methods. Paritala *et al*. (2023) has previously published an inter-user variability study on sub-001, with interval surveillance over a decade. They found the level of smoothing on segmented models to be a significant contributor to variations in aneurysm morphology, causing a 20% variation in CFD results between analysts^19^. This inter-user variability is consistent with existing studies on variations in intracranial aneurysm segmentation^24^.

While establishing universal segmentation procedures is essential to reduce variability in aneurysm morphology and subsequent analyses using this dataset, the provision of image quality metrics can support these efforts. Image quality and suitability of this dataset for identification and segmentation of IAs was thus verified through evaluation of the signal-to-noise ratio (SNR) and contrast-to-noise ratio (CNR).

**Fig. 5:**
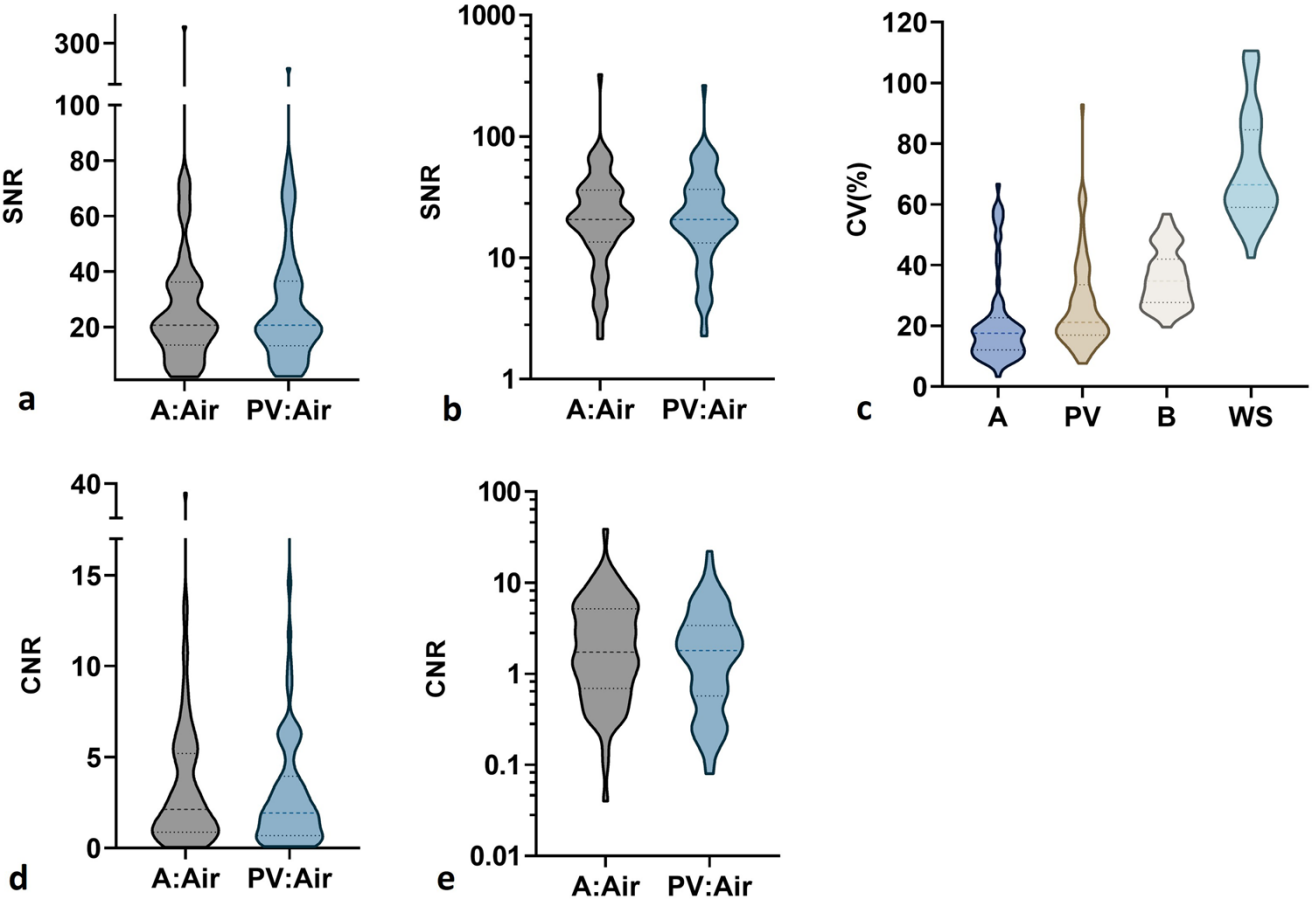
Analysis of image quality metrics across TOF-MRA patient scans (n = 73 aneurysms in 58 patients). Some patients scans (n = 5) did not include any ‘air’ region suitable for calculations, or did not include MRA annotations, and thus were excluded from analysis for all image quality metrics. (**a**) Signal-to-noise ratio (SNR) of the region of interest relative to noise in the air; (**b**) SNR presented on a logarithmic (base 10) scale; (**c**) Coefficient of variation (CV) of pixel intensity in region of interest; (**d**) Contrast-to-noise ratio (CNR) between the region of interest and a user-selected small brain region devoid of vasculature, relative to noise in the air; (**e**) CNR presented on a logarithmic (base 10) scale. A = Aneurysm, PV = Parent Vessel with Aneurysm, WS = Whole Scan, B = Brain region. Dotted lines in the violin plots indicate quartiles, with the middle being the median value.

Using the annotated masks provided by a neuroradiologist, pixel locations for the aneurysm and parent vessel were extracted to determine corresponding pixel intensities in the original scans. To evaluate contrast between the vasculature and surrounding brain tissue, pixel intensities in a user-selected small brain region devoid of vasculature was also extracted. Noise was determined based on signal intensity in the air outside of the scan. SNR, CNR and coefficients of variation (CV) were then calculated for the aneurysm-only, and aneurysm with parent vessels using equations (1) to (3) below, with results depicted in Fig. 5. Regions of interest used for calculations were sized based on the number of pixels to account for changes in imaging resolution. Where some patient scans did not include any ‘air’ regions or were annotated using DSA or CTA images (n = 5), these were excluded from analysis of all quality metrics.

SNR, signal-to-noise ratio

1 $$SNR=\frac{{\mu }_{SI,Aneurysm/Vessel}}{{\sigma }_{SI,Air}}$$

CNR, contrast-to-noise ratio

2 $$CNR=\frac{{\mu }_{SI,Aneurysm/Vessel}-{\mu }_{SI,Brain}}{{\sigma }_{SI,Air}}$$

CV, coefficient of variation (%)

3 $$CV=\frac{{\sigma }_{SI,Aneurysm/Vessel}}{{\mu }_{SI,Aneurysm/Vessel}}\times 100 \% $$

Where ${\mu }_{SI,Aneurysn/Vessel}$ is the mean signal intensity for the aneurysm-only or the aneurysm with parent vessel, ${\mu }_{SI,Brain}$ is the mean signal intensity for the neighbouring brain region, ${\sigma }_{SI,Air}$ is the standard deviation of pixel intensity in the air outside of the head, and ${\sigma }_{SI,Aneurysm/Vessel}$ is the standard deviation of pixel intensity across the aneurysm-only or the aneurysm with parent vessel.

As can be seen in Fig. 5a,d, there is a wide range of SNR and CNR values across patients, which should be considered when selecting suitable images for modelling or segmentation purposes. These have been further presented on a logarithmic scale (base 10) in Fig. 5b,c respectively for clarity due to the wide range of values.

A similar range of SNR values was previously observed across existing databases of the healthy Circle of Willis (n = 15) when comparing the signal in vessels to manual segmentations^25^. A comparable SNR range is also seen in databases containing T1-weighted images as opposed to angiograms^26^. It is uncertain as to how the quality of these images compare to those provided by Di Noto *et al*.^14^ as SNR and CNR statistics were not published alongside the dataset.

A previous study evaluated the SNR of brain arteries in the Circle of Willis using 1.5 T, 3 T and 7 T TOF-MRA scanners, finding 1.5 T scanners resulted in the lowest SNR of 23.8 which remained excellent for visualising these major arteries^27^. It has also been shown through computational simulations that for an SNR ≥ 10, vessel cross-sectional area can be accurately determined with an error of <5% for images with a sampling matrix down to 256 × 256 pixels^28^. It can be clearly seen from Fig. 5b that the SNR of most scans is greater than 10. In general, for accurate segmentation of aneurysms from the TOF-MRA scans, a high SNR is desired to ensure vasculature can be clearly differentiated from noise to produce well-defined vessel edges^29^.

A similar range of CNR values across TOF-MRA has also been reported across limited studies^30,31^. However, there is no clear guideline or evidence for a CNR range that clearly supports accurate segmentation of vasculature from TOF-MRA. From equation (2) above, a CNR > 1 indicates the contrast between the vessels and neighbouring tissue is greater than the noise itself, which is desirable for vessel segmentation. In general, a high CNR will ensure vessels are well-contrasted against the neighbouring tissue of the brain to ease segmentation^32,33^.

As illustrated in Fig. 5e, the CNR of almost half of the scans in this dataset is less than 1, implying the signal difference between the aneurysm and brain is less than the noise from the background ‘air’. Segmentation and modelling from these ‘noisy’ scans could therefore be difficult. Meanwhile, sub-004 had high CNR values of 39 for the aneurysm and 22 for the aneurysm with parent vessel, likely due to the contrast agent administered for this scan in combination with the TOF-MRA.

The IA region in most patient scans in this dataset exhibited an SNR > 10, while half had a CNR > 1, and therefore should facilitate accurate segmentations. However, many scans obtained an SNR < 10 and CNR < 1, and thus care should be taken when using these patient scans of variable image quality for further modelling and analysis. To ensure appropriate comparisons between studies utilising multiple datasets, it is important to consider these SNR and CNR results, as models developed on patient scans of high SNR may be challenging to adapt to those with a low SNR^34^.

## Usage Notes

While the free and open-source 3D Slicer software can be used for viewing and processing of raw images and clinician-segmented vascular trees, parent vessels, and IA models, the NIFTI format provided ensures various commercial software suites can also utilise the de-identified TOF-MRAs for further processing. This imaging format is also consistent with existing aneurysm repositories previously described, including the IXI database and the recently published aneurysm dataset by Di Noto *et al*. (2023). This ensures similar data-processing workflows can be used across available datasets.

Patient scans within this dataset have been collected with comparable acquisition parameters to existing datasets, albeit with some scanners using significantly larger spacing between slices, thereby increasing voxel size and reducing image resolution, as summarised in Table 1. In some cases, the voxel size is comparable to arterial diameters across the Circle of Willis, thus decreasing spatial resolution and detail. Through investigating the effect of voxel size on IA morphological and haemodynamic parameters, Berg *et al*. (2018) found a larger voxel size to result in inaccurate aneurysmal neck representation in segmentations, with the inability to capture smaller side branches, small perforators and potentially smaller aneurysms^35^. Therefore, while TOF-MRA is often used for monitoring aneurysms due to the lack of ionising radiation required and greater safety for patients, the poor spatial resolution can limit analysis of aneurysm progression and shape change. Figure 3 thus provides only an indication for whether aneurysm shape change has been observed.

Furthermore, sub-001 from this dataset has been previously investigated for inter-user variability and the effect of segmentation morphology on fluid dynamics simulations^19^. Paritala *et al*. (2023) found that minor variations in threshold segmentation parameters and the level of smoothing resulted in a 20% variation in computational fluid dynamics results related to wall shear stress and derived parameters.

Similarly, SNR and CNR is variable across the dataset as summarised in Fig. 5. and can influence segmentation accuracy and the ability to distinguish vessels from neighbouring tissue. Therefore, caution must be exercised when utilising low resolution patient scans for research necessitating segmentation and consider the inter-user segmentation variability previously described^19^. As such, voxel spacing, SNR and CNR results are provided for each scan in the supporting documentation for the dataset and can be consulted when selecting appropriate patient images to incorporate into modelling and analysis.

This broad distribution of image quality represents realistic TOF-MRA images from a major tertiary hospital, and thus is valuable for the development of robust automated patient screening and aneurysm detection algorithms. To ensure widespread use of IA models, including in frugal, remote, or austere clinical settings, it is important that clinicians and clinical research be educated and developed using TOF-MRA images of varying quality.

**Fig. 6:**
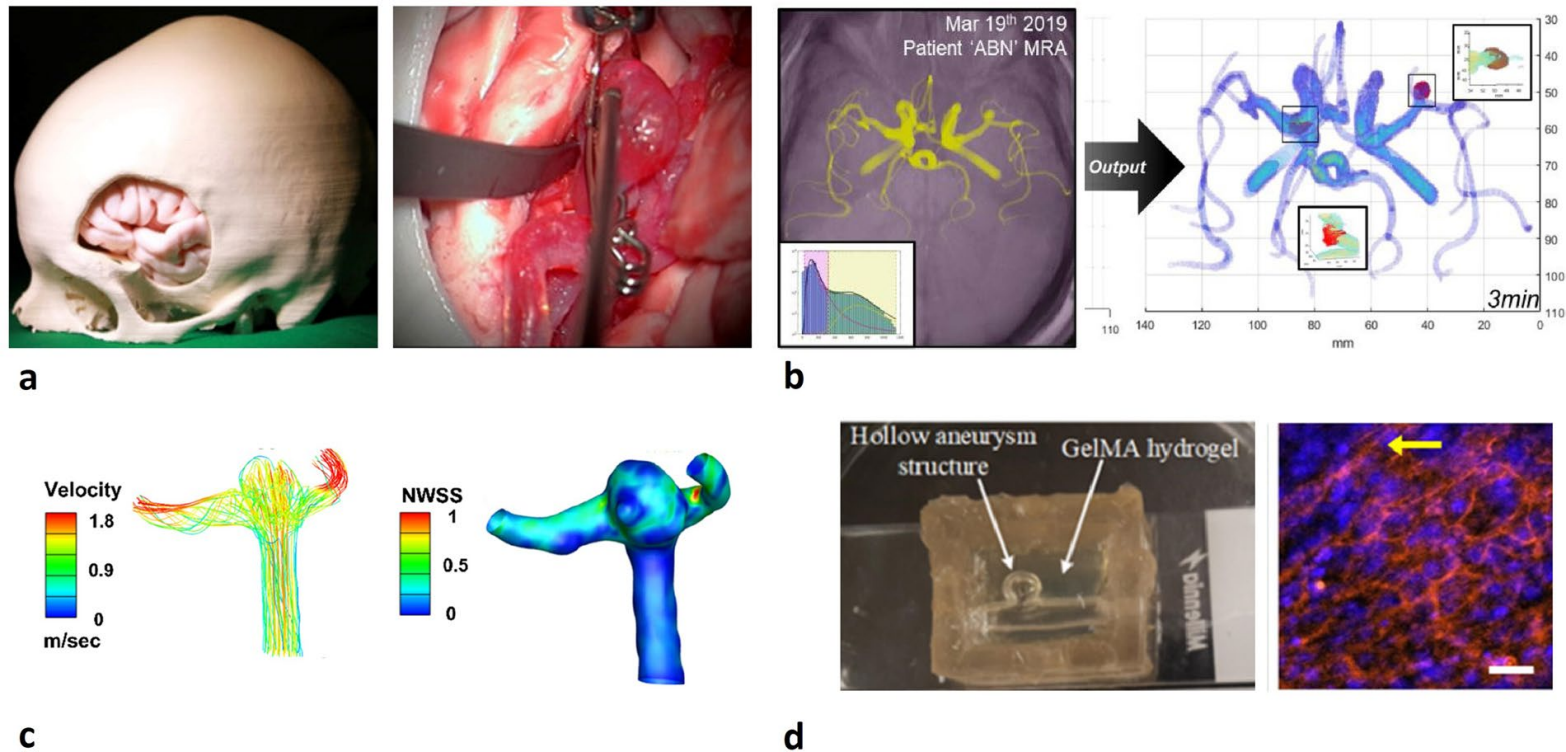
Potential applications of dataset in intracranial aneurysm research. (**a**) Patient-specific 3D printed skull, brain and middle cerebral artery aneurysm model for surgical clipping training^36^; (**b**) Automated intracranial aneurysm identification algorithm using MATLAB applied to sub-037^13^; (**c**) Computational fluid dynamics analyses of sub-001 as part of the inter-user variability study yielding velocity and wall shear stress contour plots in a middle cerebral artery aneurysm^19^; (**d**) Gelatine Methacrylate aneurysm model with open lumen, seeded with endothelial cells and smooth muscle cells^22^. Figures have been reused from referenced articles with permission and under approved licenses.

This dataset aims to support the effort to reduce bottlenecks in data availability which currently limits research and modelling into IA growth and rupture dynamics. As depicted in Fig. 6 this dataset can be, and has already been, useful for developing surgical training models and surgical guides, automated detection algorithms, in CFD studies and for evaluating cell behaviour and dynamics in *in vitro* IA models. Specifically, Fig. 6b^5^ and Fig. 6c^19^ have made use of this dataset.

## Data Availability

The described dataset was manually organised according to BIDS (v1.8.0). DICOM files were converted to NIFTI format using MRIcroGL dcm2niix software (NITRC, v1.0.20220720). Masks and annotations were prepared using 3DSlicer software (v5.2.1). Python (Spyder, v5.4.3) was used to order scans by imaging modality and scanning dates, and for the stripping of unnecessary NIFTI slices in some patients where portions of the skull not related to the Circle of Willis were also imaged. Code used for these purposes is not published as it is not required for generation or processing of the published dataset.
